# Sea Cucumber Saponins Derivatives Alleviate Hepatic Lipid Accumulation Effectively in Fatty Acids-Induced HepG2 Cells and Orotic Acid-Induced Rats

**DOI:** 10.3390/md20110703

**Published:** 2022-11-10

**Authors:** Xiaoyue Li, Beibei Zeng, Lu Wen, Yingcai Zhao, Zhaojie Li, Changhu Xue, Tiantian Zhang, Yuming Wang

**Affiliations:** 1 College of Food Science and Engineering, Ocean University of China, Qingdao 266003, China; 2 Laboratory for Marine Drugs and Bioproducts, Pilot National Laboratory for Marine Science and Technology (Qingdao), Qingdao 266237, China

**Keywords:** saponins, derivatives, cholesterol metabolism, lipogenesis, fatty acids β-oxidation

## Abstract

The sulfated echinoside A (EA) and holothurin A (HA) are two prominent saponins in sea cucumber with high hemolytic activity but also superior lipid-lowering activity. Deglycosylated derivatives EA2 and HA2 exhibit low hemolysis compared to EA and HA, but their efficacies on lipid metabolism regulation remains unknown. In this study, fatty acids-treated HepG2 cells and orotic acid-treated rats were used to investigate the lipid-lowering effects of sea cucumber saponin derivatives. Both the saponin and derivatives could effectively alleviate lipid accumulation in HepG2 model, especially EA and EA2. Moreover, though the lipid-lowering effect of EA2 was not equal with EA at the same dosage of 0.05% in diet, 0.15% dosage of EA2 significantly reduced hepatic steatosis rate, liver TC and TG contents by 76%, 41.5%, and 63.7%, respectively, compared to control and reversed liver histopathological features to normal degree according to H&E stained sections. Possible mechanisms mainly included enhancement of fatty acids β-oxidation and cholesterol catabolism through bile acids synthesis and excretion, suppression of lipogenesis and cholesterol uptake. It revealed that the efficacy of EA2 on lipid metabolism regulation was dose-dependent, and 0.15% dosage of EA2 possessed better efficacy with lower toxicity compared to 0.05% dosage of EA.

## 1. Introduction

Liver disease is one of the main causes for mortality throughout the world, due to the changes in dietary habits and lifestyle [1]. The prevalence of alcoholic fatty liver disease (ALD) and non-alcoholic fatty liver disease (NAFLD), originated from hepatic lipid accumulation, has increased dramatically and now accounts for more than 60% of all liver diseases [1,2]. In China, the alarming 29.2% prevalence of NAFLD has served as a reminder that more focus has to be given to reduce hepatic lipid accumulation [3]. So far, the recommended liver health-care advice is mainly about lifestyle improvements including healthy eating behavior and appropriate exercise, suggesting that dietary intervention to reverse the progression of hepatic lipid accumulation is an important strategy [4].

**Figure 1:**
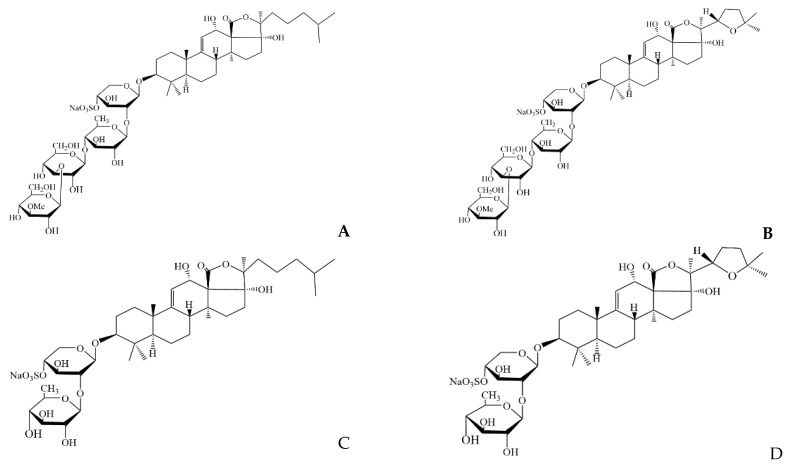
Chemical structure of sea cucumber saponins EA (**A**), HA (**B),** and their derivatives EA2 (**C**) and HA2 (**D**).

Sea cucumber, belonging to the phylum Echinodermata, is a traditional Asian food with high edible and medicinal values, which has various kinds of bioactive substances including saponins, peptides, polysaccharides, etc. Compared with terrestrial organisms, the occurrence of sulfate group in marine organisms is quite widespread, typically being reported from echinoderms represented by sea cucumber, and the sulfated substances usually exhibit excellent bioactivities [5,6]. Sea cucumber saponins also have a sulfate group, which is different from ginsenosides. A large quantity of studies have shown that sea cucumber-derived saponins exhibited considerable potential for anti-tumor, anti-diabetes, anti-obesity, and anti-hyperlipidemia effects, which have been widely used in the pharmaceutical and functional food industries [7]. It has been reported that sea cucumber species *Pearsonothuria graeffei* has relatively high content of saponins, in which holothurin A (HA) and echinoside A (EA) are two major saponins [8,9]. Though HA and EA have been proved to be efficient on regulating lipid metabolism [10], the high hemolytic activity may limit their applications [11,12]. According to our previous research, HA2 and EA2 were the main deglycosylated products by gut microflora in mice (Figure 1), and the conversion rate reached about 40% and 27%, respectively, in intestinal contents. Moreover, both the EA, HA, and their derivatives could be absorbed in intestine [13]. Additionally, the hemolysis of EA2 was remarkably reduced compared to EA [11]; however, it is yet unknown whether the sea cucumber saponins derivatives still have lipid metabolism regulating efficacy. Moreover, the comparison between saponins and deglycosylated derivatives may reveal the structure activity relationships of sea cucumber saponins.

The study was aimed to testify whether the less toxic sea cucumber saponins deglycosylated derivatives still have lipid metabolism regulating efficacy and to explore their structure activity relationships. Terrestrial ginsenoside Rh2 was used as a reference in in vitro experiments since it has been reported to exhibit remarkable ability on regulating lipid metabolism [14,15].

## 2. Results

### 2.1. Effects of Saponins on Lipid Accumulation in HepG2 Cells

To screen for appropriate modeling and experimenting concentrations, MTT assay was used to determine the non-cytotoxic concentration range of fatty acids and saponins. As shown in Figure S2A, cells treated with sea cucumber saponins, derivatives, and ginsenoside Rh2 exhibited no significance in viability at the concentration of 0.2–0.8 μM compared to normal group, but exhibited significant cytotoxic effects when the concentration increased to 1 μM. In addition, HepG2 cells did not exhibit cytotoxicity when incubated with 0–1 μM fatty acid mixture (palmitic acid: oleic acid, at 1:2 ratio) for 24 h (Figure S2B). Thus, the free fatty acids (FFAs) mixture at the concentration of 1 μM was used to induce hepatocellular lipid accumulation and the concentration of saponins was kept at 0.8 μM in the subsequent in vitro experiments.

**Figure 2:**
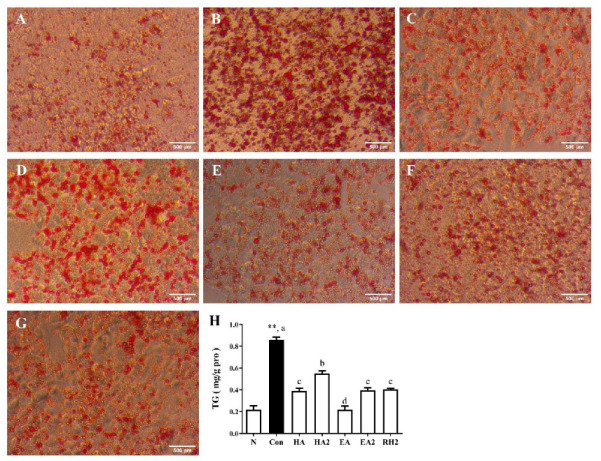
Effects of sea cucumber saponins, derivatives and ginsenoside Rh2 on lipid accumulation in HepG2 cells. Cells were treated as follows, N: serum-free DMEM containing 10% BSA; Con: serum-free DMEM containing 10% BSA and 1 μM free fatty acids (FFA); HA, HA2, EA, EA2, RH2: serum-free DMEM containing 10% BSA, 1μM FFA, and 0.8 μM saponins. After the incubation for 24 h, lipid accumulation was assessed by oil red O staining (**A**–**G**) and intracellular TG contents normalized as the amount of cellular protein (mg/g protein) (**H**). **A**: normal; **B**: control; **C**: HA; **D**: HA2; **E**: EA; **F**: EA2; **G**: RH2. Data were presented as Mean ± SEM. ** *p* < 0.01 indicates significant differences between normal and control group. Different letters represented significant differences at *p* < 0.05 among control and treated groups.

To evaluate the effects of saponins on reducing lipid accumulation in FFAs treated HepG2 cells, oil red O staining was used to observe intracellular lipid accumulation followed by the determination of intracellular TG contents. Figure 2A–G show that the number of lipid droplets was significantly higher in control group compared with that of normal group, while the number of lipid droplets in cells incubated with saponins or their derivatives was significantly reduced in comparison to control group, especially EA group, which was nearly equal to normal group. Notably, both HA2 and EA2 apparently alleviated lipid accumulation, though they were less effectively than HA and EA.

Intracellular TG contents normalized as the amount of cellular protein (mg/g protein) in Figure 2H matched the results of oil red O staining. The TG content in control group was quadrupled of normal group. In comparison to fatty acids treated group, sea cucumber saponins HA, EA, and ginsenoside Rh2 significantly reduced TG contents by 54.6%, 74.6%, and 53.0%, respectively, meanwhile sea cucumber saponins derivatives HA2 and EA2 also significantly reduced TG levels by 35.8% and 54.0%, respectively.

### 2.2. Effects of Saponins on Lipogenesis and Fatty Acid β-Oxidation in HepG2 Cells

**Figure 3:**
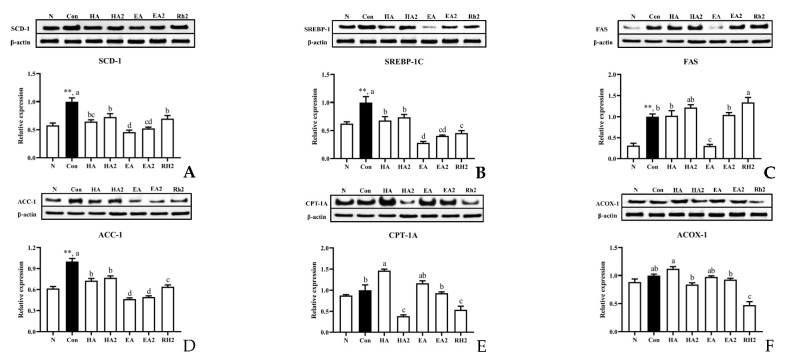
Effects of sea cucumber saponins, derivatives, and ginsenoside Rh2 on the protein expression levels of genes related to hepatic lipogenesis and fatty acid β-oxidation in HepG2 cells. Cells were treated as follows, Con: serum-free DMEM containing 10% BSA; M: serum-free DMEM containing 10% BSA and 1 μM FFA; HA, HA2, EA, EA2, RH2: serum-free DMEM containing 10% BSA, 1 μM FFA, and 0.8 μM saponins. (**A**) SCD-1, stearoyl-CoA desaturase 1; (**B**) SREBP-1, sterol regulatory element binding transcription factor 1; (**C**) FAS, fatty acid synthase; (**D**) ACC-1, acetyl-CoA carboxylase; (**E**) CPT-1A, carnitine palmitoyltransferase 1A; (**F**) ACOX-1, Acyl-CoA oxidase 1. Data were presented as Mean ± SEM. ** *p* < 0.01 indicates significant differences between normal and control group. Different letters represented significant differences at *p* < 0.05 among control and treated groups.

Based on the aforementioned results, Western blotting of several key proteins about lipid regulation was applied to comparatively explore saponins’ lipid-lowering activity mechanism further. SREBP-1 (sterol regulatory element binding transcription factor 1) is a crucial nuclear transcriptional factor involved in hepatic lipogenesis, which primarily controls the expression of lipogenic genes, such as ACC-1 (acetyl-CoA carboxylase) and FAS (fatty acid synthase). SCD-1 (stearoyl-CoA desaturase 1) also plays an important role in lipid biosynthesis by introducing the first double bond to saturated fatty acyl-CoA substrates [16]. As shown in Figure 3A–D, EA apparently downregulated the protein expressions of these four lipogenic genes mentioned above to a normal level. EA2 and Rh2 significantly reduced the expressions of SREBP-1 and ACC-1 compared to control group. In addition, the protein expressions of SREBP-1 and ACC-1 were also downregulated by HA compared to control group, while HA2 did not show off distinct reduction. In terms of fatty acid β-oxidation, Acyl-CoA oxidase 1 (ACOX1) is the first enzyme of fatty acid β-oxidation, and carnitine palmitoyltransferase 1A (CPT-1A) is the key enzyme in carnitine-transport across the mitochondrial inner membrane for long-chain fatty acids oxidation [17], so their expressions have a negative correlation with lipid accumulation. From Figure 3E,F, we can see that HA significantly increased the protein expression of CPT-1A compared to control. Ginsenoside Rh2 distinctly lowered the protein expression of CPT-1A and ACOX, and HA2 distinctly lowered the expression of CPT-1A too. Besides, EA and EA2 did not significantly alter the protein level of CPT-1A and ACOX compared to control group.

### 2.3. Effects of EA and EA2 on Liver Histopathological Features of Rats

**Figure 4:**
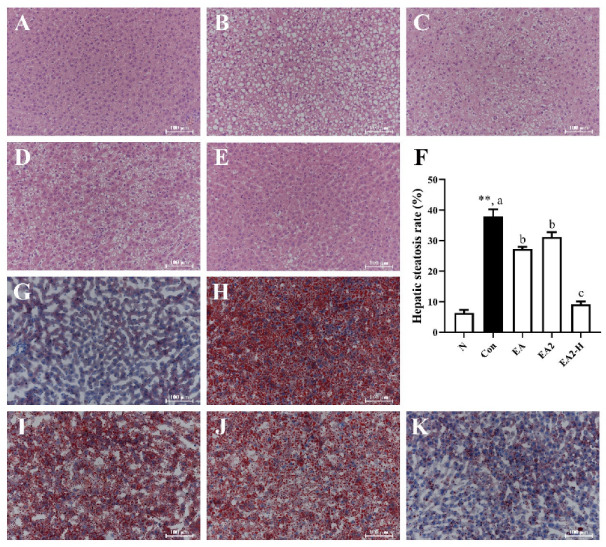
Effects of EA (0.05%), EA2 (0.05%) and EA2 -H (0.15%) on liver histopathological features and lipids deposition. Rats were fed as follows, N: normal diet; Con: normal diet supplemented with 1% orotic acid; EA, EA2: normal diet supplemented with 1% orotic acid and 0.05% EA or EA2; EA2-H: normal diet supplemented with 1% orotic acid and 0.15% EA2. Detection of liver histological features by hematoxylin-eosin (HE) staining. (**A**) N, (**B**) control, (**C**) EA (0.05%), (**D**) EA2 (0.05%), (**E**) EA2-H (0.15%). (**F**) Hepatic steatosis rate was calculated according to white areas fraction with three different views for each rat in H&E staining slices by image j. Detection of hepatic lipids accumulation by oil red O staining. (**G**) N, (**H**) control, (**I**) EA (0.05%), (**J**) EA2 (0.05%), (**K**) EA2-H (0.15%). Data were presented as Mean ± SEM. ** *p* < 0.01 indicates significant differences between normal and control group. Different letters represented significant differences at *p* < 0.05 among control and treated groups.

Liver histological features following the intervention of orotic acid (OA) and saponins containing diet (Table S2) were determined by H&E and oil red O staining as shown in Figure 4. The H&E-stained section of control group exhibited typical histopathological features with large amounts of full fat vacuoles in lobule cells and sizable quantity of inflammatory cells (Figure 4B) [18]. At the dosage of 0.05%, EA2 marginally improved the histopathological features mentioned above, but had more fat vacuoles and severer cell swelling than EA (Figure 4C,D). Interestingly, when the dosage of EA2 was increased to 0.15% (Figure 4E), the state of liver almost reversed to normal level as shown in Figure 4A. Hepatic steatosis rate was determined as white areas fraction using image j and presented in Figure 4F, which was consistent with histopathological features mentioned above. As data showed, control rats had six times higher of hepatic steatosis rate than normal group, which also indicated the successful construction of hepatic lipid accumulation. With the addition of 0.05% EA or EA2, the rates were significantly reduced by 28.1% and 17.7%, respectively, compared to control group, while 0.15% dosage of EA2 significantly decreased the rate by 76.0% and brought it close to normal (<10%). Oil red O staining represented the lipid deposition in liver (Figure 4G–K) and demonstrated the same tendency with H&E staining as described above.

### 2.4. Effects of EA and EA2 on Lipid Metabolism and Liver Injury in Rats

**Figure 5:**
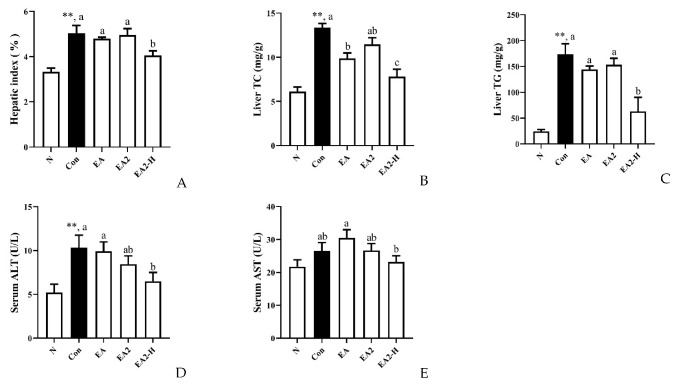
Effects of EA (0.05%), EA2 (0.05%) and EA2-H (0.15%) on hepatic lipids and liver injury. Rats were fed as follows, N: normal diet; Con: normal diet supplemented with 1% orotic acid; EA, EA2: normal diet supplemented with 1% orotic acid and 0.05% EA or EA2; EA2-H: normal diet supplemented with 1% orotic acid and 0.15%. (**A**) Hepatic index (mass ratio of liver to body); (**B**) Liver TC; (**C**) Liver TG. (**D**) Serum ALT; (**E**) Serum AST. Data were presented as Mean ± SEM. ** *p* < 0.01 indicates significant differences between normal and control group. Different letters represented significant differences at *p* < 0.05 among control and treated groups.

The physiological indexes pointing to lipid metabolism are shown in Figure 5 and Figures S3 and S4. Under the identical food intake, all the orotic acid intervened rats underwent a significant weight loss (Figure S3). We could notice that high dosage of EA2 significantly reduced the hepatic index (mass ratio of liver to body) by 19.5% compared to control, while 0.05% dosage of EA and EA2 did not show off significance with control group (Figure 5A and Figure S3). Compared to control group, EA apparently lowered hepatic TC by 26.0%, whereas high dosage of EA2 significantly reduced TC by 41.5% as shown in Figure 5B. Moreover, EA2-H group significantly decreased TG level by 56.3% compared with control group (Figure 5C). The severity of liver injury was assessed using serum ALT and AST levels as indicators of hepatocyte damage [19], and the results were shown in Figure 5D and E. The serum ALT activity was significantly doubled following the administration of OA diet and was reduced significantly by 37.1% following high dosage EA2 treatment, while EA and EA2 at 0.05% dosage did not demonstrate significant reductions compared to control group (Figure 5D). As for serum AST, saponins intervention did not exhibit significant changes from control group, while 0.15% EA2 group significantly decreased serum AST by 23.8% when compared to EA group.

Lipids metabolism in serum was shown in Figure S4. The trends of serum TG, TC, and HDL-C were comparable among groups. Normal group had the highest content while EA group had the lowest instead of control group. High dosage of EA2 could elevate the serum TG and TC significantly compared to control group. As for LDL-C, both EA2 (0.05%) and EA2-H (0.15%) groups showed a significant increasement compared to control group.

### 2.5. Effects of EA and EA2 on Lipids Excretion in Rats

**Figure 6:**
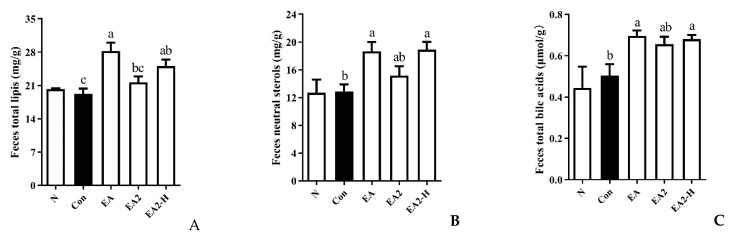
Effects of EA (0.05%), EA2 (0.05%), and EA2-H (0.15%) on fecal lipids. Rats were fed as follows, N: normal diet; Con: normal diet supplemented with 1% orotic acid; EA, EA2: normal diet supplemented with 1% orotic acid and 0.05% EA or EA2; EA2-H: normal diet supplemented with 1% orotic acid and 0.15%. (**A**) Fecal total lipids; (**B**) fecal neutral sterols; (**C**) fecal total bile acids. Data were presented as Mean ± SEM. *p* < 0.01 indicates significant differences between normal and control group. Different letters represented significant differences at *p* < 0.05 among control and treated groups.

Lipids excretion conditions after EA or EA2 intervention were compared by fecal lipids (Figure 6). EA treatment significantly elevated the total lipids content in feces by 46.9% compared to control group (Figure 6A). The total lipid content of the high-dose EA2 group was also 30.6% higher than that of the control group. Regarding the content of neutral sterols and total bile acids, EA (0.05%) significantly exceeded control group by 44.7% and 38.1%, respectively, and EA2-H (0.15%) significantly exceeded control group by 46.5% and 35.1%, respectively (Figure 6B,C).

### 2.6. Effects of EA and EA2 on the Expression of Genes Related to Fatty Acids and Cholesterol Metabolism

**Figure 7:**
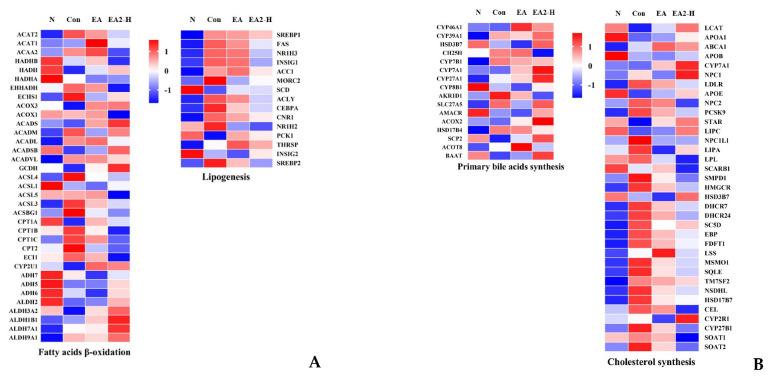
Effects of EA and EA2-H on the expressions of genes related to fatty acids metabolism (**A**) and cholesterol metabolism (**B**) according to RNA-Seq results. The gene lists were obtained from KEGG and ordered randomly.

To further study the differences of structure–activity relationship between EA and EA2, expressions of genes related to fatty acids and cholesterol metabolism were compared in Figure 7A,B. Previous studies reported that the possible mechanism of OA-induced fatty liver in rats mainly included the stimulation of triacylglycerol synthesis by SREBP1 and its target genes, inhibition of low-density lipoproteins secretion and fatty acid β-oxidation [20,21]. As shown in Figure 7A, the expression of core genes in lipogenesis such as SREBP1 and its target genes FAS and ACC1 were all upregulated by OA administration, while dietary EA2-H significantly reduced their expressions. For lipid consumption, CPT-1A gene encodes the rate-limiting enzyme of mitochondria β-oxidation pathway. We could observe that OA apparently suppressed the expression of CPT-1A, while saponins mitigated the suppression effectively. The alternations of gene expressions related to fatty acids metabolism were consistent with experimental findings in HepG2 cells described above as well as mechanisms reported by Wang et al. [21]. As for the inhibition of low-density lipoproteins secretion, the expressions of apolipoprotein-coding gene APOA1, APOB, and APOE showed in Figure 7B indirectly reflected the tendency that saponins could elevate the inhibition of low density lipoproteins secretion caused by OA intervention, particularly high dosage EA2 [22]. In addition to altered genes discussed above, we surprisingly found that sea cucumber saponins could alleviate OA-induced hepatic lipids accumulation by the increasement of lipids excretion, especially through the transformation from cholesterol to bile acids. The rate-limiting step in the synthesis of primary bile acids was reportedly controlled by CYP7A1 (cytochrome P450 Family 7 Subfamily A Member 1) [23]. Results showed that the expression of CYP7A1 was evidently upregulated after treatment with EA or EA2, particularly high dosage EA2 (Figure 7B). Moreover, the majority of genes in this pathway exhibited same tendency with CYP7A1, indicating that sea cucumber saponins, especially high dosage of EA2, could increase the primary bile acids synthesis and hence enhance the excretion of cholesterol.

## 3. Discussion

The results of oil red O staining and intracellular TG contents in HepG2 cells indicated that EA had the best performance in preventing lipid accumulation followed in order by HA, EA2 and Rh2 at around the same degree. Though HA2 showed to be less effective, it nevertheless significantly reduced the lipid accumulation in HepG2 cells. Interestingly, saponins derived from sea cucumber outperformed that from ginseng on alleviating lipid accumulation. Moreover, HA and EA exhibited superior ability on reducing intracellular TG content than their respective derivatives HA2 and EA2.

Protein expressions of key genes related to hepatic lipogenesis and fatty acid β-oxidation revealed that EA played the best role in restraining lipogenesis among all the saponins followed by EA2. A reduction in FAS and SREBP1 protein content was closely followed by a decrease in TG content in HepG2 cells. It was widely reported that SREBP1 was an important transcriptional regulator of lipogenesis, and FAS played an important role in lipogenesis [24]. HA played the best role in promoting oxidation followed by EA and EA2. Taking together, EA and its derivative EA2 presented superior efficacy on reducing lipids accumulation in HepG2 cell model, and they were chosen to conduct in vivo experiments subsequently. Though the capacity of Rh2 to reduce lipids accumulation had been approved above, the protein expressions of lipogenesis and β-oxidation did not change as anticipated. Previous research reported that Rh2 dosage-dependently reduced adipogenesis and downregulated the protein expression of FAS compared to DMSO control in 3T3-L1 cells during the differentiation [14]. However, the current investigation found that FAS protein expression was elevated. We speculated that the discrepancy between cell lines was the reason.

Additionally, there were a few minor discrepancies on genes expression between cell protein and RNA-Seq of rat liver. For example, SREBP1 RNA expression of EA was equal with control in rat liver while the protein expression was higher in HepG2 cell, and ACOX1 RNA expression was lower than control in rat liver while the protein expression was about equal with control in HepG2 cell. Despite the contrasts being present, we could see more related genes expression changes in RNA-Seq, and the overall effect represented by RNA-Seq supported that EA2-H played a better role in hepatic lipid lowering effect compared to EA. The conflicts might attribute to the differences between a complex organism and a simplified cell line. Differences between cell line and rats might be based on the degree of dependence on fat, which acts as a major energy resource. HepG2 generated from hepatocellular carcinoma was dependent less on fat substrates due to Warburg effect compared to the rats [25].

**Figure 8:**
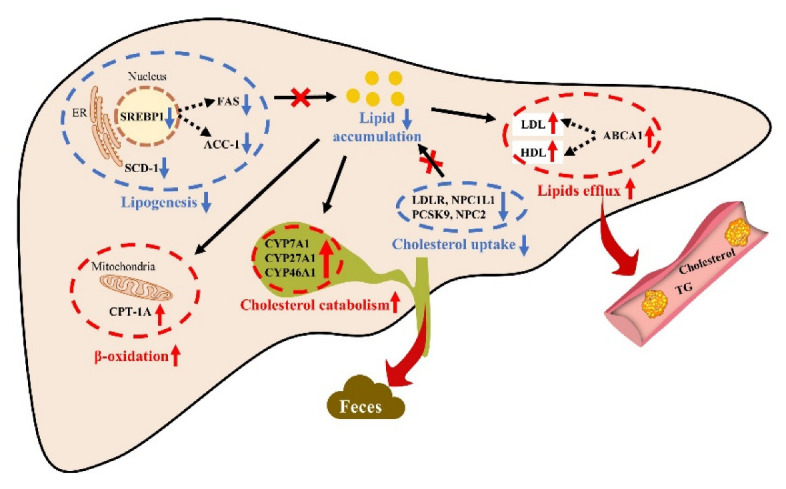
Possible mechanisms of sea cucumber saponins and their derivatives on alleviating hepatic lipid accumulation in fatty acids-treated HepG2 cells and orotic acid-treated rats.

As shown in Figure 5B,C, only EA2-H significantly reduced hepatic TC and TG, while EA could significantly reduce HepG2 cellular TG content compared to EA2 at same dosage of 0.8 μM in Figure 2H. The two results were not contradictory, especially the coincident tendency that 0.05% EA2 exceeded 0.05% EA a bit in liver TC and TG content. Surprisingly, the efficacy loss of EA2 compared to EA at same dosage of 0.05% could be compensated by 0.15% dosage of EA2 as indicated by Figure 5B,C. The rats treated with OA exhibited fatty livers and subnormal levels of cholesterols and triglycerides in blood [26], which was contrary to traditional high fat diet-induced hepatic lipid accumulation with increased blood lipids. Figure 5B,C and Figure S4 showed that EA2-H could significantly downregulate hepatic lipids levels and upregulate serum lipid levels, while EA significantly downregulated serum TG level. Combining with genes alternations shown in Figure 7B, including the downregulation of LDLR, APOB (involved in LDL-c uptake from blood), and NPC1L1 (involved in cholesterol absorption from intestinal lumen); the restriction of PCSK9 and NPC2 (mediating cholesterol uptake intracellular); the promotion of ABCA1 and APOA1 (mediating cholesterol efflux to blood); and the suppression of LPL and SCARB1 (mediating plasma lipids clearance), the acting differences between EA and EA2 could be summarized as EA was more effective at plasma lipids clearance, and EA2 was more powerful in restriction of hepatic cholesterol uptake both from blood and peripheral tissue such as intestine [27]. Alterations in the lipid composition of the feces indicated that EA increased the flux to excretion more compared with EA2. Except for the genes alternations on cholesterol metabolism discussed above, genes related in fatty acids β-oxidation and lipogenesis, which had been studied at protein level in HepG2 cells, represented by Figure 7A, could elaborate the structure–activity relationship of EA and EA2. As for fatty acids β-oxidation, the rate-limiting CPT1A was more upregulated by EA but not EA2, which was consistent with Figure 3E. The contradictory changes in CPT2 and CPT1 families might be attributed to the complex feedback regulations. Acyl-CoA dehydrogenase series (ACADs) were more upregulated by EA2, while the next reactions participated by HADHs and ACATs were suppressed, presumably it was due to the complex regulatory mechanism of the reversible reaction. Surprisingly, ADHs and ALDHs (participated in detoxification of lipid peroxidation) were promoted more by EA2, and the reason needs to be further studied. On the other hand, lipogenesis related genes exhibited uniform changes that EA2-H restrained more than EA, and the difference between cellular protein levels might be attributed to dosage increasement as mentioned before. To sum up, EA acts more than EA2 on promotion of fatty acids β-oxidation and plasma lipids clearance, while EA2 acts more than EA on restriction of hepatic cholesterol uptake and promotion of cholesterol catabolism to bile acids, and 0.15% EA2 could restrain lipogenesis more powerful than single dosage of EA. Figure 8 demonstrated that the effectiveness of sea cucumber saponins and their derivatives on reducing hepatic lipid accumulation was attributed to the restriction of lipogenesis and cholesterol uptake, the promotion of fatty acids β-oxidation, cholesterol catabolism to bile acids, and lipids excretion.

Results above showed that 0.15% dosage of EA2 could compensate for the efficacy loss due to deglycosylation compared with EA at same 0.05% dosage. Moreover, according to our previous studies, 0.05% EA in diet was approximately equal to 0.3 μg/mL whole blood based on its absorption characteristics, and the hemolysis rate of 0.3 μg/mL EA was about 40%, while the hemolysis rate of EA2 under same concentration was only about 6% compared to erythrocytes in saline. Notably, the hemolysis rate of EA2 remained less than 10% until the concentration up to 20 μg/mL, whereas the hemolysis rate of EA changed from 60% to 100% during the concentration range of 5–20 μg/mL. It indicated that one possible way for removing hemolysis-induced application restrictions on sea cucumber saponins was the preparation of derivatives.

## 4. Materials and Methods

### 4.1. Materials

Sea cucumber (*Pearsonothuria graeffei*) was purchased from Nanshan aquatic products market (Qingdao, Shandong, China). Ginsenoside Rh2 (97%) was obtained from Shanxi ZhongXin Biotechnology Co., Ltd. (Xi’an, Shanxi, China). The human liver hepatocellular carcinoma cell line (HepG2) cells were purchased from Shanghai Institute of Biochemistry and Cell Biology (Shanghai, China). Dulbecco’s Modified Eagle’s Medium (DMEM) and Fetal bovine serum (FBS) were purchased from Biological Industries (Kibbutz Beit-Haemek, Israel). Fatty-acid-free bovine serum albumin (BSA), palmitic acid, oleic acid, oil red O dyes, and 3-(4, 5- dimethylthiazol-2-yl)-2,5-diphenyltetrazolium bromide (MTT) were purchased from Beijing Solarbio Science & Technology Co., Ltd (Beijing, China). Primary antibodies against SREBP-1, FAS, ACC-1, CPT1, ACOX1, SCD-1, β-actin, and secondary antibodies were purchased from Abcam, Inc. (Cambridge, MA, USA). Protein ladders (10–180 kDa) were purchased from Yeasen Biotechnology Co., Ltd. (Shanghai, China). Triglyceride (TG), total cholesterol (TC), aspartate aminotransferase (AST), alanine aminotransferase (ALT), high-density lipoprotein cholesterol, low-density lipoprotein cholesterol, and total bile acid (TBA) assay kits were all purchased from Nanjing Jiancheng Bioengineering Institute (Nanjing, Jiangsu, China).

### 4.2. Preparation of Sea Cucumber Saponins and Their Derivatives

Crude sea cucumber saponins were isolated from air-dried sea cucumber (*P. graeffei*) body walls using the previously reported method [28]. Briefly, ethanol extractions were evaporated in vacuo and then partitioned with water and chloroform. The water layer was separated and extracted with n-butanol. After concentrating, the extracts were loaded in an HP20 resin column and eluted with water, 80% ethanol, and 100% ethanol in sequence. The fraction eluted with 80% ethanol was collected as crude saponins. Crude saponins were then loaded on ODS silica gel (YMC-Pack, Japan) column chromatography and washed by stepwise elution with methanol and water to collect pure HA and EA.

Based on the previous work of chemical structure identification and content determination by Dong et al., the purity of EA and HA was determined with high performance liquid chromatography-ultraviolet detector [29]. Concisely, Eclipse XDB-C18 column (150 × 4.6 mm, 5 μm) from Agilent (Palo Alto, CA, USA) and UV detection at 205 nm were used for the determination of their content. The column was kept at 30 °C. The mobile phase consisted of a mixture of acetonitrile (A) and 0.1% ammonium bicarbonate (B). The flow velocity of mobile phase was 1 mL/min, and the composition was as follows: 0–5 min, 30% A; 5–30 min, 60% A; 30–35 min, 30% A, and the injection volume was 10 μL.

Then, EA and HA were separately hydrolyzed by pectinase Pectinex Ultra SP-L according to Chen et al. with slight modification [11]. Briefly, saponins were dissolved in sodium acetate solution (pH 4.6) with enzyme activity of 100 U at 40 °C. After the incubation for 72 h, the solids were collected by centrifugation at 11,300 g for 10 min at room temperature. High performance liquid chromatography–mass spectrometry (HPLC-MS) was used for the examination with Agilent Eclipse XDB-C18 column (150 × 4.6 mm, 5 μm) according to our previous reports on structure and content analysis of sea cucumber saponins derivatives [13]. The mobile phase consisted of a mixture of 5 mM ammonium formate (A) and acetonitrile (B). For EA and EA2, the gradient elution was as follows: 0–1 min, 50% A; 1–12 min, 50–38% A; 12–13 min, 38–50% A. For HA and HA2, the gradient elution was as follows: 0–1 min, 60% A; 1–8 min, 60–43% A; 8–9 min, 43–60% A. Mass spectrometric experiments were performed on an Agilent G6410 Triple Quad (QQQ) tandem mass spectrometer (Palo Alto, USA) in negative ESI ion mode. Operation conditions were applied as follows: capillary voltage, 3.5 kV; atomizing voltage (N2), 40 psi; ion source temperature, 300 °C; drying gas (N2) flow rate, 10.0 L/min; drying gas temperature, 350 °C. The single ion monitoring (SIM) parameters was shown in Table S1. Chemical structures of sea cucumber saponins EA (PubChem CID: 156832), HA (PubChem CID: 20056045) and their derivatives EA2, HA2 were shown in Figure 1, and the purities were all confirmed to be over 90% as shown in Figure S1.

### 4.3. Experimental Design

Palmitic acid and oleic acid-induced lipid accumulation HepG2 cell model was first utilized to compare the effects of sea cucumber saponins and their corresponding deglycosylated derivatives on inhibiting lipid accumulation. Before the formal experiment began, MTT assay was used to determine the non-cytotoxic concentration range of fatty acids and saponins; then certain concentrations of fatty acids and saponins were applied to treat HepG2 cells and evaluate lipid lowering effects by oil red O staining and intracellular TG contents (assay kits). Moreover, the possible underlying mechanism was explored preliminarily by Western blotting of several key lipid metabolism regulatory genes including SREBP-1, ACOX, SCD-1, ACC-1, CPT-1A, FAS.

Further in vivo validation experiments with low dosage (0.05%) of EA or EA2 and triple dosage (0.15%) of EA2 were designed with orotic acid-treated Wistar rats, due to the superior effects of EA and EA2 on reducing lipids accumulation in vitro. During animal feeding, food intake and body weight were monitored regularly. Then, tissues, blood, and feces were collected for subsequent analysis. H&E and oil red O staining of liver were applied for histological features observation, and the hepatic steatosis rate was determined by white areas fraction in H&E staining slices by image j. To evaluate the extent of hepatic lipids accumulation and explore the possible regulatory mechanisms, physiological indexes pointing to lipid metabolism, including lipids distribution in serum, liver, and feces were measured with corresponding assay kits. Finally, RNA-Seq of liver was utilized for further mechanism exploration.

### 4.4. Cell Culture

HepG2 cells were purchased from Shanghai Bioleaf Biotech Co., Ltd. (Shanghai, China) and maintained in serum-containing medium (DMEM containing 10% fetal bovine serum (FBS), 100 IU/mL penicillin and 0.1 mg/mL streptomycin) in incubator with a humidified atmosphere (5% CO_2_) at 37 °C. The medium was usually changed every 2 days until cells reached to the confluence of 80–90%. Then, the cells were digested and sub-cultured into 6 or 96 well cell culture plates at the density of 4.2 × 10^4^ cells/cm^2^ by 0.25% trypsin containing 0.02% EDTA for the following experiments. 

### 4.5. Cell Viability Analysis

The viability of HepG2 cells was measured by MTT assay method [30]. Briefly, the HepG2 cells were seeded as described above in a 96-well culture plate and incubated in the incubator for 24 h to reach around 80% confluence, then the cells were treated with fresh serum-free DMEM containing HA, HA2, EA, EA2, and Rh2 at 0.2, 0.8, and 1 μM for another 24 h, or containing palmitic acid and oleic acid (1:2 ratio) at 0.3, 0.5, 0.8, and 1 μM for 24 h. Finally, the medium was gently removed and 200 μL of MTT (0.5 mg/mL) was added into each well for further incubation. After incubating at 37 °C for 4 h, the supernatant was carefully replaced with 150 μL DMSO to dissolve the crystal. The absorbance at 570 nm was measured using a microplate reader (Tecan spark 10M, Männedorf, Switzerland). Cell viability was expressed as a percentage of viable cells obtained relative to that of controls.

### 4.6. Oil Red O Staining

The hepatocellular steatosis inducing medium was prepared based on the previous report with slight modification [31,32]. Generally, palmitic acid and oleic acid were separately dissolved in 0.1 M sodium hydroxide solution and incubated at 70 °C for 1 h. Then, the fatty acid sodium solution was mixed together with serum-free DMEM containing 10% fatty-acid-free BSA as required proportion. Finally, HepG2 cells were treated with palmitic acid and oleic acid (1:2 ratio, 1 μM) to induce an in vitro hepatic steatosis model with lipid accumulation. HepG2 cells were divided into seven groups, namely normal, control, HA, HA2, EA, EA2, and Rh2 groups, respectively. The concentration of free fatty acids (FFAs) and saponins were kept at 1 μM and 0.8 μM, respectively.

After 24 h incubation, the cells were washed with 1×PBS buffer twice, and subsequently fixed with 4% paraformaldehyde for 20 min. Then, the cells were washed with 60% isopropanol for 5 min before staining with freshly prepared oil red O solution (oil red O dye and water at a ratio of 3:2) for 30 min at room temperature. After 30 min, oil red O solution was immediately removed and cells were washed with PBS three times. Finally, cells were observed with PBS under microscope (Carl Zeiss, Oberkochen, Germany).

### 4.7. Western Blotting Analysis

The HepG2 cells were collected and lysed using RIPA lysate containing 1 mM protease inhibitor phenyl methane sulfonyl fluoride (PMSF) and phosphatase inhibitor complex on ice for 20 min. Then, the lysates were centrifuged in 4 °C at 12,000 rpm for 5 min and the supernatant was obtained as protein sample used in subsequent analysis. The protein was separated on sodium dodecyl sulfate–polyacrylamide gel electrophoresis (SDS-PAGE) after measuring the protein concentration by a bicinchoninic acid (BCA) protein assay kit. The protein was transferred onto a polyvinylidene fluoride (PVDF) membrane followed by blocking in a buffer containing 5% BSA for 2 h. The blots were separated and incubated with primary antibodies ab28481 SREBP-1 (97 kDa), ab184032 ACOX (72 kDa), ab236868 SCD-1 (37 kDa), and ab8226 β-actin (42kDa); ab45174 ACC-1 (265 kDa), ab234111 CPT-1A (88 kDa), ab133619 FAS (45 kDa), and β-actin (42 kDa) at 4 °C overnight, then incubated with specific secondary antibodies at room temperature for 2 h. Finally, the blots were visualized using an ECL reagent chemical imaging system (Tanon-5200, shanghai, China) and the gray scale of the band was analyzed.

### 4.8. Animals and treatments

All animal procedures were performed in accordance with the Guidelines for Care and Use of Laboratory Animals of Ocean University of China and approved by the Animal Ethics Committee of the College of Food Science and Engineering, Ocean University of China. Wistar rats were purchased from Vital River (Beijing, China) and housed individually with a 12 h light/dark cycle, a constant temperature of 23 ± 2 °C and humidity of 65  ±  15%. After a 7-day adaption period, 35 rats were randomly divided into five groups (n = 7) according to the body weight, including normal group (normal diet as shown in Table S2), control group (normal diet supplemented with 1% OA), EA group (normal diet supplemented with 1% OA and 0.05% EA), EA2 group (normal diet supplemented with 1% OA and 0.05% EA2) and EA2-H group (normal diet supplemented with 1% OA and 0.15% EA2) [33]. Rats were allowed free access to water and food for 10 days. In the last three days, feces were collected, lyophilized, and weighed for subsequent analysis. At the end of experimental period, the rats were sacrificed by bleeding from the abdominal aorta after a 12 h overnight fasting. Serum was collected from blood by centrifugation at 4000 g (4 °C) for 10 min and stored at −80 °C. Livers were quickly excised, weighed, and then fixed for histopathological analysis or quick-frozen with liquid nitrogen and stored at −80 °C until analysis.

### 4.9. Histological Analysis

For H&E staining, livers were collected and fixed in 4% paraformaldehyde solution, then embedded in paraffin, sectioned at 5 μm, and stained with hematoxylin and eosin. As for oil red O staining, frozen livers were sectioned at 10 μm and placed on slides, fixed in 4% paraformaldehyde, and then dyed with oil red O. Slices were observed with a light microscope (Ni-E, Nikon, Japan). Image j was utilized to determine the white area (fat component) fraction with three different views for each rat in H&E staining slices.

### 4.10. Lipids Extraction

Total lipids in liver and feces were extracted by homogenization with chloroform-methanol mixture (2:1, *v/v*) according to the modified method of Folch [34,35]. The crude extracts were mixed with one-fifth of its volume of saline solution (0.15 M NaCl). After blending, the mixtures were placed into a separatory funnel to collect the bottom chloroform phase. Then, chloroform solutions were condensed by vacuum for use.

### 4.11. Determination of Lipids in Liver and Serum

Triglyceride (TG), total cholesterol (TC), aspartate aminotransferase (AST), alanine aminotransferase (ALT), high-density lipoprotein cholesterol, and low-density lipoprotein cholesterol in serum were measured under the instruction of assay kits. Liver TC and TG contents were measured using assay kits with total lipids extracted above.

### 4.12. Determination of Lipids in Feces

Neutral sterols and bile acids were determined with modified method as previously described [36,37]. Briefly, total lipids extractions were hydrolyzed in 20% KOH (ethylene glycol) for 20 min, and neutral sterols were extracted from the alkaline mixture by petroleum ether. The extracted hydrolysate was then acidified, and bile acids were extracted with diethyl ether. Analysis of the sterol derivatives were performed on a GC Agilent 6890 Series (Agilent Technologies, Santa Clara, CA, USA) using a capillary column HP-5 (30 × 0.32 mm and 0.25 mm film thickness; Agilent Technologies) with internal standard 5α-cholestane. Total bile acids were measured using enzymatic reagent kits.

### 4.13. RNA-Seq Transcriptome Analysis

Total RNA was extracted from liver of each rats using Trizol reagent, then the RNA from control, EA, and EA2-H rats was mixed equally according to group sets (seven samples from the same group were mixed into one), thereafter the samples were sent to Shanghai Majorbio Bio-Pharm Biotechnology Co., Ltd. (Shanghai, China) for RNA quality examination, library construction, and sequencing according to instructions (Illumina, San Diego, CA, USA). The raw data was trimmed and quality controlled by SeqPrep (https://github.com/jstjohn/SeqPrep, accessed on 20 November 2020) and Sickle (https://github.com/najoshi/sickle, accessed on 20 November 2020) with default parameters. The expression level of each transcript was calculated according to the transcripts per million reads (TPM) method. Gene abundance was quantified by RSEM (http://deweylab.biostat.wisc.edu/rsem, accessed on 20 November 2020).

### 4.14. Statistical Analysis

Data analysis was performed with Statistical Product and Service Solutions (SPSS) version 18.0 software (SPSS Institute, Chicago, IL, USA), and the results were presented as mean ± standard error of mean (SEM) of triplicate experiments. Statistically significant differences among control and saponins treated groups were assessed by one-way ANOVA and Tukey’s test. Comparisons between normal and control groups were assessed by Student’s *t*-test. *p* < 0.05 was regarded statistically significant.

## 5. Conclusions

In conclusion, when at same dosage, the derivatives of sea cucumber saponins did not work as well as saponins, but also exhibited excellent ability on reducing lipid accumulation in HepG2 cell model. Western blotting of several key proteins in lipogenesis and fatty acids β-oxidation revealed that HA primarily promoted fatty acids β-oxidation, while EA and EA2 could restrain lipogenesis and promote fatty acids oxidation simultaneously, which might account for their superior efficacy on reducing lipids accumulation in HepG2 cell model. In vivo experiment proceeded in orotic acid-treated rats was applied to further investigate the efficacy of EA2 on alleviating hepatic lipid accumulation. The efficacy of EA2 on lipid metabolism regulation was dose-dependent, and 0.15% dosage of EA2 exhibited better lipid-lowering efficacy and lower hemolytic activity compared to 0.05% dosage of EA although deglycosylated derivatives EA2 did not serve as good as EA at same 0.05% dosage. Along with the restriction of lipogenesis and hepatic cholesterol uptake, the promotion of fatty acids β-oxidation, cholesterol transformation to bile acids, and lipids excretion also played an important role for sea cucumber saponins to achieve lipid-lowering activity in vivo. The obtained results clarified that sea cucumber saponins derivatives could exhibit superior efficacy on lipid metabolism regulation by dosage increasement meanwhile with lower toxicity compared to saponin EA. Given the promising findings, more studies need to be performed to explore other decomposed or derived forms of sea cucumber saponins on lipid regulation or other fields. The development of derivatives may provide a strategy for safe and wide utilization of sea cucumber saponins to avoid hemolysis limitation as a source of natural bioactive components.

## Data Availability

Not applicable.

## Supplementary Materials

The following supporting information can be downloaded at: https://www.mdpi.com/article/10.3390/md20110703/s1, Figure S1: Analysis of sea cucumber saponins EA, HA and their derivatives EA2, HA2 by liquid chromatogram; Figure S2. Cytotoxic effects on HepG2 cells after 24 h incubation with gradient concentration of saponins (A) and free fatty acids (B); Figure S3. Effects of EA (0.05%), EA2 (0.05%), and high-dosage EA2 (0.15%) on food intake and body weight change in Wistar rats fed with experimental diets; Figure S4. Effects of EA and EA2 on serum lipids. A: Serum TG; B: Serum TC; C: Serum HDL-C; D: Serum LDL-C; Table S1. Detection and identification of prepared saponins and their derivatives; Table S2. Compositions of experimental diets.
