# Supplementary material for: Sea Cucumber Saponins Derivatives Alleviate Hepatic Lipid Accumulation Effectively in Fatty Acids-Induced HepG2 Cells and Orotic Acid-Induced Rats
Source: Mar Drugs. 2022 Nov 10;20(11):703. doi: 10.3390/md20110703 (PMC9697935; doi:10.3390/md20110703)
Supplement: Supplementary file 1 [file marinedrugs-20-00703-s001.zip › marinedrugs-1907813-supplementary.pdf]

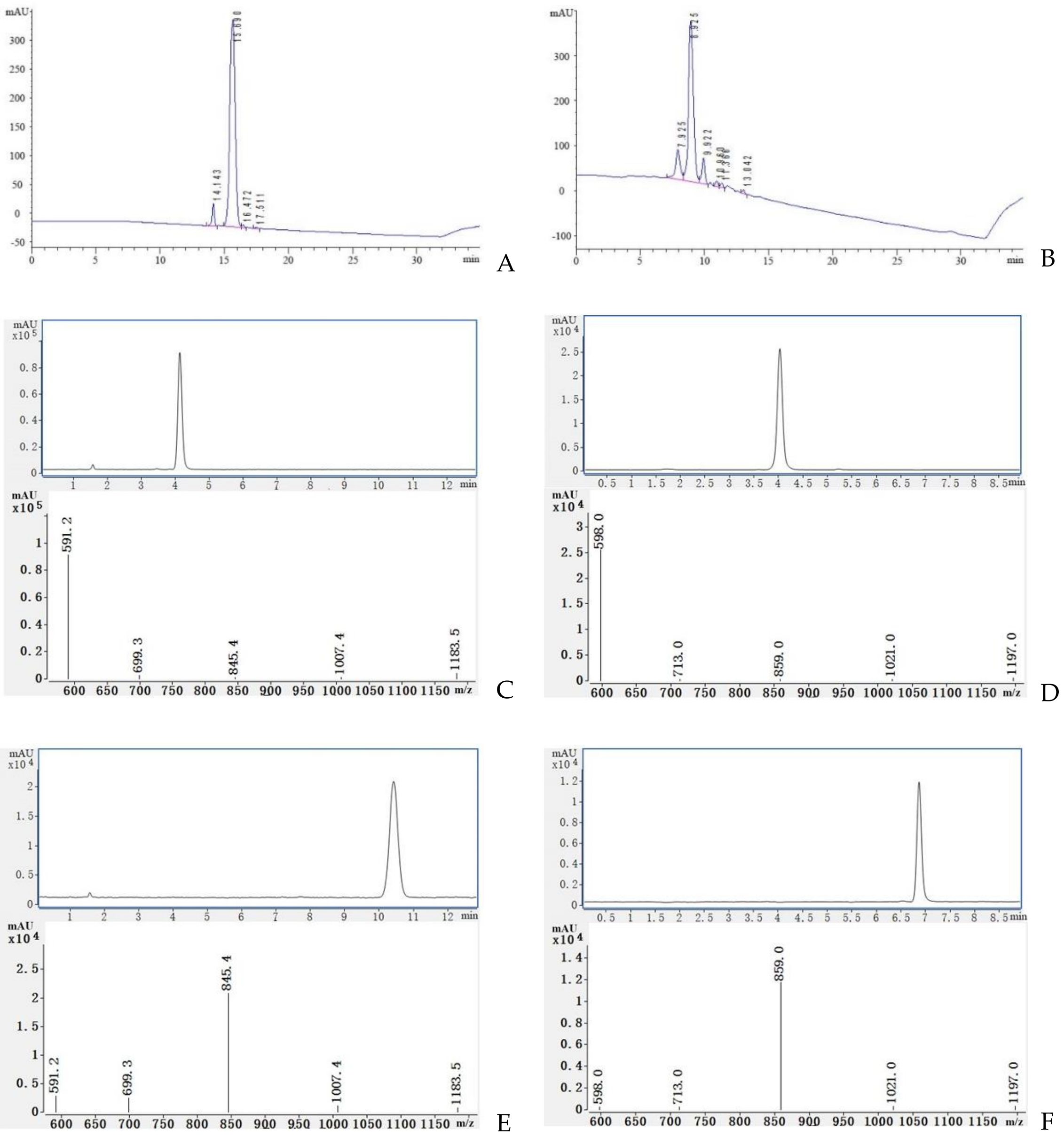

Figure S1. Analysis of sea cucumber saponins EA, HA and their derivatives EA2, HA2 by liquid chromatogram. Purity analysis of EA (A) and HA (B) by HPLC. Total ion chromatography and mass spectrogram of sea cucumber saponins EA (C) and HA (D) as well as corresponding metabolites EA2 (E) and HA2 (F) by LC-MS.

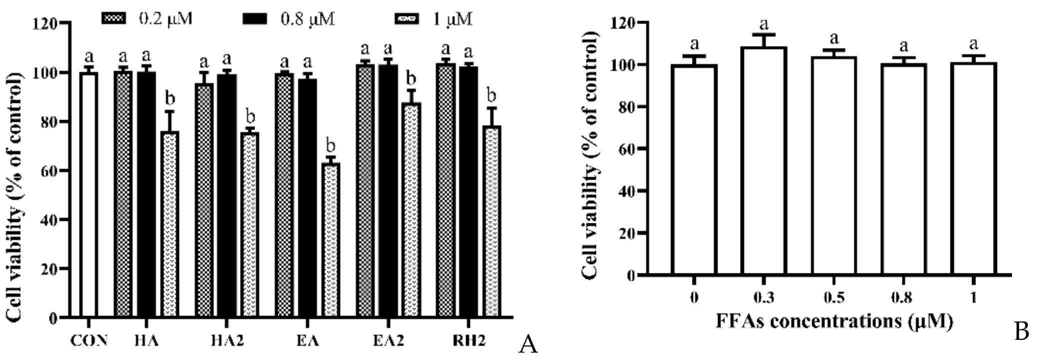

Figure S2. Cytotoxic effects on HepG2 cells after 24h incubation with gradient concentration of saponins (A) and free fatty acids (B). The free fatty acids (FFAs) were mixed with palmitic acid (PA) and oleic acid (OA) at a ratio of 1:2, and the concentration was calculated by total. Data were obtained from three independent experiments and were presented as Mean ± SEM. Different letters represented significant differences at $P < 0.05$ among treated groups.

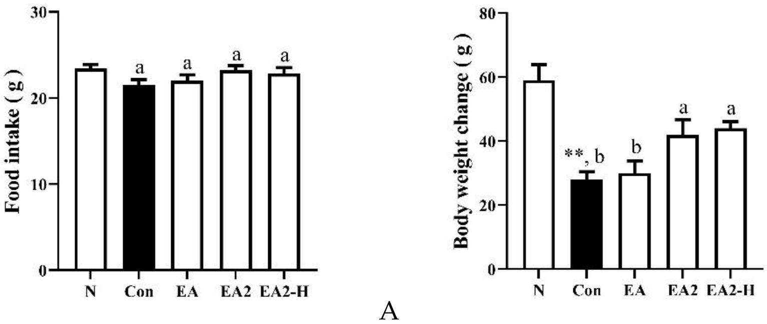

A B

Figure S3. Effects of EA (0.05%), EA2 (0.05%), and high-dosage EA2 (0.15%) on food intake and body weight change in Wistar rats fed with experimental diets. A: Food intake; B: Body weight change. Data were presented as Mean ± SEM. ** $P < 0.01$ indicates significant differences between normal and control group. Different letters represented significant differences at $P < 0.05$ among control and treated groups.

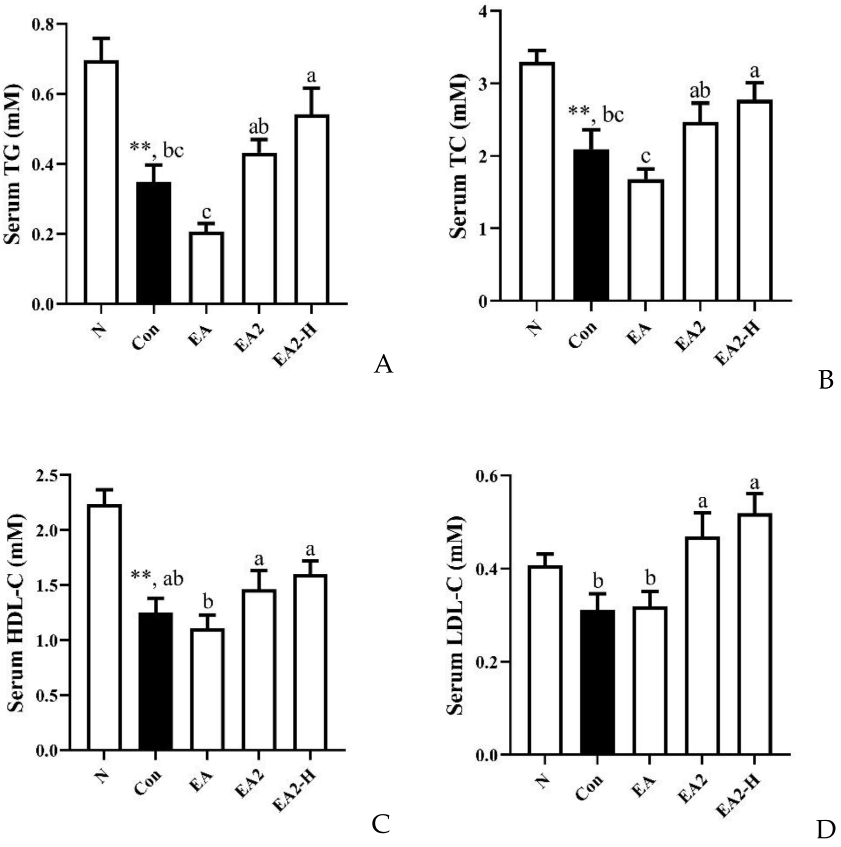

Figure S4. Effects of EA and EA2 on serum lipids. A: Serum TG; B: Serum TC; C: Serum HDL-C; D: Serum LDL-C. Data were presented as Mean ± SEM. ** $P < 0.01$ indicates significant differences between normal and control group. Different letters represented significant differences at $P < 0.05$ among control and treated groups.

Table S1. Detection and identification of prepared saponins and their metabolites.

Name	Molecular mass	Precursor ion	Formula	Retention time (min)
EA	1183.522	$[M-2H]^{2-}$	$C_{54}H_{86}O_{26}SH$	4.2
HA	1197.499	$[M-2H]^{2-}$	$C_{54}H_{84}O_{27}S$	4.0
EA2	845.400	$[M-H]^{-}$	$C_{41}H_{65}O_{16}SH$	10.4
HA2	859.38	$[M-H]^{-}$	$C_{41}H_{62}O_{17}S$	6.8

Table S2. Compositions of experimental diets.

Ingredients (g/kg)	Normal	Control	EA	EA2	EA2-H
Casein	200	200	200	200	200
Cornstarch	499.5	489.5	489	489	488
Sucrose	100	100	100	100	100
Corn oil	100	100	100	100	100
Mineral mix	35	35	35	35	35
Vitamin mix	10	10	10	10	10
Choline bitartrate	2.5	2.5	2.5	2.5	2.5
Cellulose	50	50	50	50	50
DL-methionine	3	3	3	3	3
Orotic acid	-	10	10	10	10
EA	-	-	0.5	-	-
EA2	-	-	-	0.5	1.5
